# Pleural metastases from differentiated thyroid cancer: clinical features and long-term outcomes

**DOI:** 10.1530/ETJ-24-0147

**Published:** 2025-01-29

**Authors:** Mengyue Liu, Juan Tang, Nan Sun, Chuang Xi, Chentian Shen, Song Hongjun, Quanyong Luo, Xianzhao Deng, Zhongling Qiu

**Affiliations:** ^1^ Department of Nuclear Medicine, Shanghai Sixth People’s Hospital Affiliated to Shanghai Jiao Tong University School of Medicine, Shanghai, China; ^2^ Department of Pathology, Shanghai Sixth People’s Hospital Affiliated to Shanghai Jiao Tong University School of Medicine, Shanghai, China; ^3^ Department of General Surgery, Sixth People’s Hospital Affiliated to Shanghai Jiao Tong University School of Medicine, Shanghai, China

**Keywords:** pleural metastases, malignant pleural effusion, radioiodine avidity, differentiated thyroid cancer

## Abstract

**Objective:**

Pleural metastasis (PM) is rare in patients with differentiated thyroid cancer (DTC). Radioiodine (^131^I) therapy has been the main treatment for postoperative metastasis and recurrence of DTC. However, clinical data on PM from DTC are limited. This study investigated the clinicopathological characteristics of patients with PM from DTC that were treated surgically and with ^131^I therapy and evaluated their long-term prognosis and prognostic factors.

**Methods:**

A review of the Shanghai Sixth People’s Hospital medical records from 2010 to 2023 identified PM in 27 of 14,473 patients with DTC. Overall survival (OS) was assessed by the Kaplan–Meier method.

**Results:**

The prevalence of PM in DTC was 1.87‰ (27/14,473). The median age at the time of initial diagnosis of PM was 59 years (range: 34–79). At the end of follow-up, eight patients (29.63%) had disease progression (PD), four (14.81%) had a partial response, and the remainder had stable disease; no patient achieved complete response. Twelve patients (44.44%) died, and 15 (55.56%) survived. Thirteen patients (48.15%) did not show ^131^I avidity, and 16 (59.26%) had radioiodine-refractory DTC (RR-DTC). Twenty patients (74.07%) had malignant pleural effusion (MPE), which was large in 11 cases (40.74%) and moderate in two. More-than-moderate MPE (*P* = 0.031), lack of ^131^I avidity (*P* = 0.041) and RR-DTC (*P* = 0.030) were significantly associated with worse 5-year OS in patients with PM of DTC.

**Conclusions:**

PM is rare in DTC. Lack of ^131^I avidity, RR-DTC and more-than-moderate MPE are associated with poor OS in patients with DTC and PM.

## Introduction

Approximately 586,000 cases of differentiated thyroid cancer (DTC) were reported in 2020 worldwide, making DTC the ninth most common incident cancer (1). Papillary thyroid cancer (PTC) is the most common type of DTC and accounts for approximately 80% of all DTCs (2). Patients with DTC tend to have a good prognosis, with a 10-year overall survival (OS) rate of over 95% (3). However, 5–20% of these patients can develop distant metastasis during the course of the disease, leading to a less favorable prognosis (4).

The prognosis of metastatic DTC varies according to the location of distant metastasis, with the lungs and bones being the major sites. However, extensive metastases to the lung, bone, central nervous system and occasionally other organs can be observed (5). In rare cases, DTC may metastasize to the pleura, but clinical data on pleural metastasis (PM) are limited. To date, fewer than 20 reports of PM from thyroid cancer have been published, 10 (76.92%) of which have been in patients with PTC (6, 7, 8, 9, 10, 11, 12, 13).

Historically, the main treatment for DTC has been curative surgery at the time of initial diagnosis and radioiodine (^131^I) for metastatic or recurrent DTC after thyroidectomy. However, up to 15% of patients develop radioiodine-refractory DTC (RR-DTC) and require systemic treatment (14). Lenvatinib and sorafenib have been recommended by US Food and Drug Administration as the first-line treatments for patients with progressive RR-DTC and have significant benefits (2). Until April 2022, lenvatinib, sorafenib and anlotinib had been approved by China’s National Medical Products Administration for the treatment of progressive RR-DTC (15).

PM from thyroid cancer has been reported only occasionally and mainly as case reports or small case series (6, 7, 8, 9, 10, 11, 12, 13, 16, 17, 18). To our knowledge, there is no consensus on the treatment for PM of DTC, and the efficacy of treatment options remains unclear. In this study, we investigated the clinical features, long-term outcomes and independent prognostic factors for OS in patients treated for PM of DTC.

## Materials and methods

### Study participants

The Nuclear Medicine Department is the main referral center for ^131^I treatment in China. We retrospectively analyzed the medical records of 14,473 patients who received ^131^I treatment after total or near-total thyroidectomy for DTC at the Sixth People’s Hospital Affiliated to Shanghai Jiao Tong University School of Medicine between 2010 and 2023. After the exclusion of patients for whom complete data were unavailable, those with other malignant tumors and those with poorly differentiated thyroid cancer, 27 patients with PM of DTC were enrolled in the study. The investigation was approved by the local ethics committee of Shanghai Sixth People’s Hospital. Depending on when PM was detected, patients were categorized as having metastases at presentation (detected before or within 6 months after thyroidectomy) or delayed distant metastases (detected more than 6 months after thyroidectomy) (19).

### Diagnostic criteria for PM of DTC

Definitive cytological or pathological confirmation of PM was not available for all patients. Diagnosis of PM was confirmed using either of the following two criteria (20): a ^131^I-avid lesion detected in the chest by a therapeutic ^131^I-whole-body scan (^131^I-WBS) that was suggestive of PM on ^131^I single-photon emission computed tomography/computed tomography (^131^I-SPECT/CT) images or no uptake by metastatic lesions in the chest on therapeutic ^131^I-WBS but a positive result (maximum standardized uptake value ≥2.5) in pleural nodules on ^18^F-fluorodeoxyglucose positron emission tomography/computed tomography (^18^F-FDG-PET/CT) and an elevated serum thyroglobulin (Tg) concentration (defined as >1 ng/mL during suppression of thyroid-stimulating hormone (TSH) or >10 ng/mL after stimulation of TSH) (21).

### Diagnosis criteria for pleural effusion

Pleural effusion was classified as small, moderate or large based on quartile ranking in the anteroposterior direction and the greatest anteroposterior depth recorded along the midclavicular line. This classification system defines effusions as small if they fall within the first anteroposterior quartile, moderate if within the second quartile and large if within the third or fourth quartile. For indeterminate cases, the anteroposterior dimension is the determining factor, with effusions being classified as small if the measurement is <3 cm and moderate if it is <10 cm at maximum extension (22). During follow-up, the extent of pleural effusion and changes therein were evaluated by CT.

### Diagnostic criteria for RR-DTC

RR-DTC was evaluated according to the 2015 American Thyroid Association guidelines, which define structurally evident RR-DTC using the following criteria (23): i) primary and/or metastatic lesions do not absorb ^131^I, ii) primary and/or metastatic lesions lose the ability to concentrate ^131^I after previous evidence of uptake, iii) some lesions have a measurable ^131^I level while others do not and iv) progression of primary/metastatic disease within 1 year despite substantial uptake of ^131^I.

### Treatment and follow-up

All patients were required to adhere to a low-iodine diet and not take levothyroxine for at least 3–4 weeks before ^131^I treatment. Conventional assessments were performed before administration of ^131^I, including serum thyroid hormone (FT3, FT4 and TSH) concentrations, serum Tg and anti-Tg antibody (TgAb) levels and neck ultrasound and chest CT scans. The empiric prescribed activity of ^131^I was determined. An oral ^131^I dose of 3.7 GBq (100 mCi) was administered in patients with uncertain distant metastasis status, while those with confirmed PM received an oral ^131^I dose in the range of 5.55–7.4 GBq (150–200 mCi); 3–5 days later, patients underwent ^131^I-WBS with ^131^I-SPECT/CT fusion imaging. The interval between the two therapies ranged from 6 to 12 months. Physicians could opt to administer additional ^131^I treatment based on the specific clinical scenario and cumulative ^131^I activity. ^131^I treatment was stopped in patients with DTC and PM that were not radioiodine-avid following ablation of residual thyroid tissue, with some patients who had progression transitioning to molecular targeted therapy, such as tyrosine kinase inhibitors (TKIs). These patients underwent conventional assessments of FT3, FT4 and TSH concentrations and serum Tg and TgAb levels every 6 months. All patients underwent CT scans annually. If necessary, ^18^F-FDG-PET/CT scans were performed to confirm clinical status during follow-up. OS was assessed at the end of follow-up as the interval between the initial detection of PM of DTC and death from any cause.

### Evaluation of therapeutic effect

RECIST (Response Evaluation Criteria in Solid Tumors) 1.1 in combination with Tg changes was used to evaluate the therapeutic efficacy of PM of DTC, which has been used in some studies for distant metastases (5, 24). RECIST version 1.1 was used to evaluate the response to treatment based on anatomical imaging of metastases during follow-up (25); the baseline value was determined as the lesion size measured on imaging at the time of initial diagnosis of PM. Efficacy was categorized as progressive or non-progressive disease. Non-progressive disease includes complete response (CR), partial response (PR) and stable disease (SD). Progressive disease (PD) was defined as a 20% increase in metastatic volume. CR was defined as disappearance of metastatic pleural nodules, and PR was defined as a 30% reduction in lesion volume. SD was defined as neither meeting the criteria for PR nor those for PD.

### Statistical analysis

Continuous variables are expressed as the median and standard deviation or range, and categorical variables are expressed as the number (percentage) as appropriate. OS rates were assessed using the Kaplan–Meier method. Statistical analyses were performed using SPSS version 26.0 (IBM Corp., USA; https://www.ibm.com/products/spss-statistics) and Prism version 7.0 (GraphPad Software Inc., USA; https://www.graphpad.com/features). A *P*-value <0.05 was considered statistically significant.

## Results

### Clinicopathological characteristics of patients with PM from DTC

Twenty-seven of 14,473 patients diagnosed with DTC during the study period had histologically and radiologically confirmed PM and were included in this analysis. The characteristics of these 14 DTC patients are summarized in Tables 1, 2, 3. The incidence of PM from DTC was 1.87‰ (27/14,473) with 12 confirmed by the puncture pathology of pleural effusion. Fifteen (55.56%) of the 27 patients were female, and 12 (44.44%) were male. Eight patients (29.63%) were younger than 55 years, and 19 (70.37%) were aged 55 years or older. Twenty-two patients had PTC, and five had follicular thyroid carcinoma. Only three (11.11%) of the 27 patients presented with a solitary PM, and 24 (88.89%) had metastases to other organs (lung: *n* = 20 [74.07%]; bone: *n* = 9 [33.33%]; lung and bone: *n* = 7 [25.93%]; and mediastinum: *n* = 9 [33.33%]). All 27 patients received ^131^I treatment following thyroidectomy. The median number of ^131^I treatments was two (range: 1–11), and the median dose was 472 mCi (range: 100–2100). Seven patients received doses higher than 600 mCi. Nine patients also received TKIs treatment (sorafenib: *n* = 5; anlotinib: *n* = 4).

**Table 1:** Characteristics and related data of patients with pleural metastasis from DTC.

Patient characteristics					Other synchronous distant metastases	Detecting modality	Tumor characteristics			TKIs
#	Sex	Age	HIST	Years			#	Localization	Diameter (cm)	
1	F	59	PTC	12	Lungs	^18^F-FDG-PET/CT	2	Right	Pleural thickening	Sorafenib
2	F	72	FTC	0.25	Lungs, bones, pericardium	^131^I-SPECT/CT	1	Right	1.5	-
3	F	68	PTC	0.16	Lungs, bones, mediastinum	^18^F-FDG-PET/CT	5	Left	1.0	-
4	F	51	PTC	0	Lungs, brain, mediastinum, soft tissue	^131^I-SPECT/CT	1	Right	2.4	Anlotinib
5	M	75	PTC	10	Lungs, bones, mediastinum	^18^F-FDG-PET/CT	2	Left	2.5, 4.2	Anlotinib
6	M	44	PTC	3.6	Lungs	^18^F-FDG-PET/CT	1	Left	1.3	-
7	F	61	PTC	11	Lungs, bones, mediastinum	^18^F-FDG-PET/CT	1	Right	1.0	-
8	M	79	FTC	16.5	Mediastinum, bones	^18^F-FDG-PET/CT	7	Right	7.0	Sorafenib
9	F	51	PTC	26.5	Lungs	^131^I-WBS	1	Left	1.6	-
10	F	56	PTC	1.2	Lungs, mediastinum	^18^F-FDG-PET/CT	2	Bilateral	1.3, 3.5	Anlotinib
11	M	47	PTC	11	Lungs	^18^F-FDG-PET/CT	8	Left	1.2–4.4	Sorafenib
12	F	60	PTC	0.33	Lungs, bones, mediastinum, abdominal wall	^18^F-FDG-PET/CT	3	Bilateral	L:1.3; R:1.7, 3.0	Anlotinib
13	M	59	PTC	3	Peritoneum	^18^F-FDG-PET/CT	1	Left	Pleural thickening	-
14	M	65	PTC	2.8	Lungs, bones, mediastinum, brain	^18^F-FDG-PET/CT	1	Left	1.6	Sorafenib
15	F	65	PTC	6.8	Lungs, muscle group	^18^F-FDG-PET/CT	2	Left	1.9, 2.0	-
16	F	34	FTC	0.25	-	^18^F-FDG-PET/CT	1	Right	0.4	-
17	F	62	PTC	0.75	Lungs	^131^I-SPECT/CT	2	Left	2.4, 3.7	-
18	M	55	PTC	17.2	Lungs	^131^I-SPECT/CT	1	Right	2.7	-
19	F	54	PTC	4.25	Lungs, liver	^131^I-SPECT/CT	5	Bilateral	1.2–1.9	-
20	M	58	PTC	9.4	Lungs, pericardium	^131^I-SPECT/CT	1	Right	Pleural thickening	-
21	M	60	PTC	8	-	^131^I-SPECT/CT	2	Right	Pleural thickening	-
22	M	55	PTC	2.8	-	^18^F-FDG-PET/CT	3	Bilateral	1.2	-
23	F	62	FTC	3.6	Lungs, bones	^18^F-FDG-PET/CT	3	Bilateral	5.5	Sorafenib
24	F	70	FTC	10	Bones	^18^F-FDG-PET/CT	4	Right	4	-
25	M	50	PTC	6	Lungs	^18^F-FDG-PET/CT	3	Bilateral	Pleural thickening	-
26	M	74	PTC	3	Lungs, bones	^18^F-FDG-PET/CT	3	Bilateral	Pleural thickening	-
27	F	34	PTC	0	Bones	^18^F-FDG-PET/CT	6	Right	3.3	-

DTC, differentiated thyroid cancer; M, male; F, female; age, age of initial diagnosis of pleural metastasis from DTC; HIST, histology; PTC, papillary thyroid cancer; FTC, follicular thyroid cancer; years, years after total thyroidectomy; ^131^I-SPECT/CT, ^131^I-single-photon emission computed tomography/computed tomography; ^18^F-FDG-PET/CT, ^18^F-FDG-positron emission tomography/computed tomography; ^131^I-WBS, ^131^I-whole-body scan; TKIs, tyrosine kinase inhibitors.

**Table 2:** ^131^I uptake, activity and follow-up details of the patients.

Pt #	^131^I uptake	CIA (mCi)	PE	Confirmation modality	Tg^1^ (ng/mL)	Tg^2^ (ng/mL)	FUT, months	Follow-up	Cause of death
1	No	200	S	FU+I	146.90	19.70	0.23	Alive	-
2	Yes	200	No	FU+I	283.00	30.40	4.9	Alive	-
3	Yes	800	BL, S	FU+I	4000.00	59.90	12	Alive	-
4	No	200	S	FNAB	36.00	411.00	11	Dead (12 months after Dx)	Cerebrovascular accident
5	No	200	BL, S	FU+I	444.00	582.00	10.17	Alive	-
6	No	350	S	FU+I	183.00	13.50	1.57	Alive	-
7	Yes	600	No	FU+I	170.80	2.40	13	Alive	-
8	Yes	2100	L	Puncture	100.37	20,031.00	14.33	Dead (14 months after Dx)	Mediastinum metastases
9	Yes	375	No	FU+I	17.30	1.54	22.63	Alive	-
10	No	250	L	Puncture	12,443.00	19,70.00	25.33	Alive	-
11	No	300	S	FNAB	90.50	25,000	31.87	Alive	-
12	Yes	300	BL, L	Puncture	1168.00	8432.00	48.17	Alive	-
13	No	150	L	Puncture	67.40	170.00	4	Dead (4 months after Dx)	Peritoneum metastases
14	No	350	No	FU+I	1168.00	1174.00	49.13	Alive	-
15	Yes	300	S	FU+I	94.60	7510.00	49.33	Dead (49 months after Dx)	Lung metastases
16	Yes	100	S	FNAB	553.00	5.35	50.23	Alive	-
17	Yes	400	No	FU+I	584.00	3.03	55.37	Alive	-
18	Yes	730	L	Puncture	150.40	455.70	4	Dead (4 months after Dx)	MPE
19	Yes	600	L	Puncture	271.00	308.40	101.5	Dead (105 months after Dx)	MPE
20	Yes	700	L	Puncture	114.70	18,936.00	6	Dead (6 months after Dx)	MPE
21	Yes	350	L	Puncture	77.80	104.00	164.9	Dead (164 months after Dx)	MPE
22	No	400	No	FU+I	278.00	495.00	0.17	Alive	-
23	Yes	200	No	FNAB	220.80	9.90	2.42	Alive	-
24	No	2000	L	Puncture	271.70	300.52	1.33	Dead (16 months after Dx)	MPE
25	No	200	M	Puncture	882.70	17,443.00	0.33	Dead (4 months after Dx)	MPE
26	No	200	M	Puncture	89.23	19,925.00	0.67	Dead (8 months after Dx)	Progressive bone metastases
27	No	200	L	Puncture	176.60	337.00	0.33	Dead (4 months after Dx)	MPE

CIA, cumulative ^131^I activity; FU+I, follow-up + imaging; FUT, follow-up time; FU, follow-up; Pt #, patient number; PE, pleural effusion; BL, bilateral; S, small; M, moderate; L, large; FNAB, fine-needle aspiration biopsy; MPE, malignant pleural effusion; Tg^1^, Tg levels 6 months after 131I treatment; Tg^2^, Tg levels at the end of follow-up.

**Table 3:** Pleural metastases from thyroid cancer previously reported in the literature.

Reference	Publication year	Age/sex	Pathology	Diagnostic methods	Localization of PM	Other distant metastases	Years	FU	Other Symptoms	PE
Chen *et al.* (6)	2022	43/F	PTC	Biopsy, ^68^Ga-FAPI PET/CT	L	-	9	NA	Dyspnea, cough	+
Capron *et al.* (16)	2019	77/M	ATC	Biopsy	L (multiple)	Mediastinum	9	NA	Dyspnea	Large
Kosmas *et al.* (7)	2018	69/F	PTC	Cytologic, ICC	R	-	1	Died 4 months after Dx	Dyspnea	+
Kim *et al.* (8)	2017	61/F	PTC	Biopsy, ICC	R	Lungs	19	Died 4 months after Dx	-	+
Sakamoto *et al.* (9)	2015	64/F	PTC	Cytology	L	Multiple bilateral neck, lungs	29	Died later that year	-	Moderate
		82/F	PTC	Cytology	R (multiple)	Parathyroid, mediastinal	8	Died the next year	Neck mass, weight loss, early satiety	Moderate
		50/F	PTC	Cytology	R	Pituitary, lungs, liver, ribs, pelvis	3	Died 4 years after Dx	Abdominal pain, vomiting, diarrhea	−
Noda *et al.* (10)	2014	79/M	PTC	Thoracoscopy	L	Recurrence 4 years after initial primary Dx	12	NA	-	Small
Rosenstengelet *et al.* (11)	2013	71/M	PTC	Thoracoscopy, biopsy	R	Lungs	11	NA	Dyspnea	Large
Uchida *et al.* (17)	2019	73/F	ATC	Autopsy	R	-	-	Died 17 months after Dx	Dyspnea	Large
Bagherzadegan *et al.* (18)	2009	63/F	HTC	Thoracentesis	L	Lungs	16	NA	Dyspnea	Large
Qiu & Luo (12)	2009	32/M	PTC	Biopsy	L	Lungs and bones	-	NA	-	−
Siddaraju *et al.* (13)	2007	46/M	PTC	Biopsy	L	-	-	NA	Breathlessness, thyroid swelling, PE	−

M, male; F, female; PTC, papillary thyroid cancer; FTC, follicular thyroid cancer; ATC, anaplastic thyroid cancer; HTC, Hürthle thyroid cancer; ICC, immunocytochemistry; Dx, diagnosis of pleural metastasis from thyroid cancer; years, years after detection of primary tumor; NA, not available; PE, pleural effusion.

### Radiological detection of PM and pleural effusion

Patients with PM from DTC were rarely symptomatic. Shortness of breath was observed in 10 patients (37.03%), and dull chest pain was observed in six (22.22%). PM was detected by ^131^I-WBS with ^131^I-SPECT/CT in 11 patients and by ^18^F-FDG PET/CT in 16. Ten patients had one metastatic site, five had two metastatic sites, and nine had three or more metastatic sites. The maximum number of PMs was eight. Twenty patients (74.07%) also had pleural effusion, which was considered large in 11 cases (40.74%), moderate in two and small in seven. Bilateral PM was detected in four cases. Patients with massive pleural effusion had varying degrees of cough and dyspnea.

### Response to ^131^I treatment and TKIs for PM

**Figure 1:**
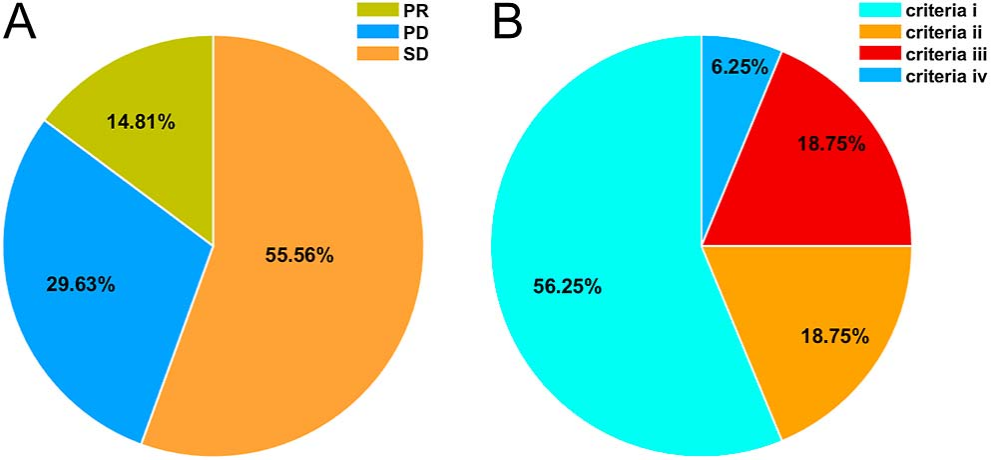
Disease status at the end of follow-up (A) and categories of RR-DTC (B).

In these patients, 14 of the 27 patients showed ^131^I avidity and 13 did not. Sixteen (59.26%) cases were categorized as RR-DTC, and 11 (40.74%) were categorized as non-RR-DTC. RR-DTC was confirmed by criterion i in nine of the 16 patients, criterion ii in three, criterion iii in three and criterion iv in one (Fig. 1B). Three of the 14 patients who had ^131^I avidity were diagnosed with RR-DTC on the basis of subsequent repeated ^131^I treatment (criterion ii: *n* = 1; criterion iii: *n* = 2).

Final disease status was assessed at the end of follow-up. Eight (29.63%) occurred as PD, four (14.81%) occurred as PR, 15 (55.56%) occurred as SD, and no patient achieved CR (Fig. 1A). In 14 patients with ^131^I uptake, four occurred as PD, three occurred as PR and seven occurred as SD. In 13 patients with non-^131^I avidity, four occurred as PD, one occurred as PR, and eight occurred as SD. In 16 patients diagnosed with RR-DTC, seven occurred as PD, one occurred as PR, and eight occurred SD. In 11 patients with non-RR-DTC, one occurred as PD, three occurred as PR, and seven occurred as SD.

Nine patients with progressive RR-DTC were treated with TKIs. Five patients received sorafenib treatment: three occurred as SD (cases 1, 14 and 23) and two occurred as PD (cases 8 and 11). Cases 1, 14 and 23 were treated for 11, 2 and 6 years, respectively, and presented SD. Case 8 diagnosed at 79 years old was treated for seven courses and eventually achieved PD, while case 11 with eight pleural nodules was treated for just 1 month and achieved PD. Four patients received anlotinib treatment: two occurred as SD (cases 4 and 5), one occurred as PR (case 10), and one occurred as PD (cases 12). Cases 4 and 5 were treated for 4 months and 1 year, respectively, and occurred as SD. Case 10 occurred as PR after 6 months of anlotinib. Case 12 experienced PD after one course of RIA treatment. However, after 8 months of anlotinib, a Tg reduction and shrinkage of lesions were observed.

### Serum Tg changes

**Figure 2:**
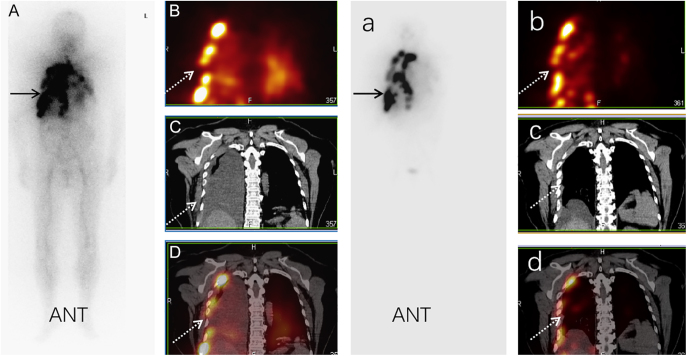
Representative case with a PR. The patient was a 51-year-old woman (case 3) who underwent near-total thyroidectomy with radical right neck dissection for PTC followed by ^131^I therapy at a dose of 7.4 GBq (200 mCi) for pulmonary metastases. A ^131^I-WBS performed 5 days after oral administration of ^131^I revealed diffuse uptake in the chest (A, solid black arrow). A ^131^I-SPECT/CT was then performed to localize the lesions in the chest and indicated uptake of ^131^I in multiple pleural and lung nodules, combined with severe MPE (B, C, D). The patient was retreated with 7.4 GBq of ^131^I for multiple metastases 4 months after the first ^131^I treatment. Post-treatment ^131^I-WBS and ^131^I-SPECT/CT fusion images showed markedly less ^131^I uptake in multiple pleural nodules, indicating a reduction in the lung nodules and MPE (B, C, D). At the same time, the stimulated Tg level decreased from 11,553.00 ng/mL to 1142.00 ng/mL. The patient achieved a PR after four cycles of ^131^I therapy (total dose, 29.6 GBq).

**Figure 3:**
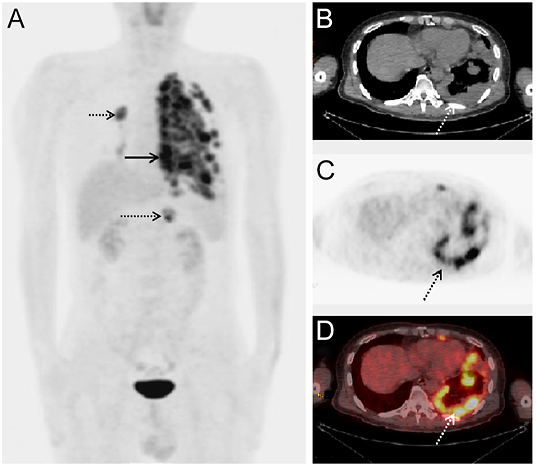
Representative case with PD. The patient was a 47-year-old man (case 11) who underwent total thyroidectomy with radical left neck dissection for PTC and was referred for ^131^I treatment of the postsurgical thyroid remnant and metastases. A ^131^I-WBS performed 5 days after oral administration of 7.4 GBq ^131^I did not show intense uptake. However, the serum Tg level increased to >25,000.00 ng/mL. Therefore, a ^18^F-FDG-PET/CT was performed to evaluate non-^131^I-avid lesions. The maximum intensity projection image (A) showed intense ^18^F-FDG uptake in lesions in the right chest (A, black short-dotted arrow) that were compatible with those in the left chest (A, black solid arrow) and abdomen (A, black long-dotted arrow). The increased activity seen on axial CT (B), PET (C) and fused PET/CT (D) scans indicated that ^18^F-FDG uptake was located in the left pleural region (B, C, D, dotted arrow), indicating PM. Although the patient is still alive, his disease has progressed and his Tg has remained >25,000.00 ng/mL persistently in the TSH suppressed status.

We compared Tg levels measured 6 months after ^131^I therapy with the final levels measured at the end of follow-up. The mean Tg level increased from 325.60 ng/mL (range: 67.40–1168.00) to 14,680.91 ng/mL (range: 170.00–25,000.00) in the eight patients with PD, decreased from 4395.00 ng/mL (range: 553.00–12,443.00) to 509.57 ng/mL (range: 3.03–1970.00) in the four with PR and increased from 264.00 ng/mL to 283.00 ng/mL in the remaining 15 patients with SD. A representative patient with a PR (case 3) is shown in Fig. 2, and a representative patient with progressive disease (case 11) is shown in Fig. 3.

### Survival analysis and prognostic factors for PM of DTC

**Figure 4:**
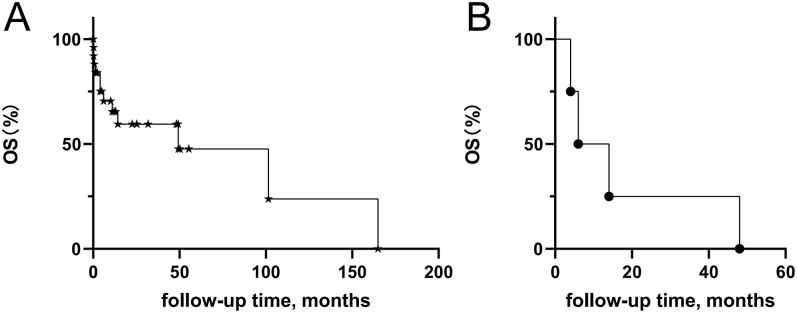
Kaplan–Meier curves for OS in patients with PM from DTC in our present cohort (A) and in the literature (B).

The median follow-up duration was 25.37 months (range: 0–164.9) with a mean of 11 months. At the end of follow-up, 12 patients (44.44%) died and 15 (55.56%) survived. Nine patients died within the second year from diagnosis of PM (cases 4, 8, 13, 18, 20, 24, 25, 26 and 27). Regarding the remaining three patients, cases 15, 19 and 21 died in the 49th, 105th and 164th month after diagnosis, respectively. Causes of death are summarized in Table 2. The 1-year, 3-year and 5-year OS rates were 74.07, 66.67 and 62.96%, respectively (Fig. 4).

**Figure 5:**
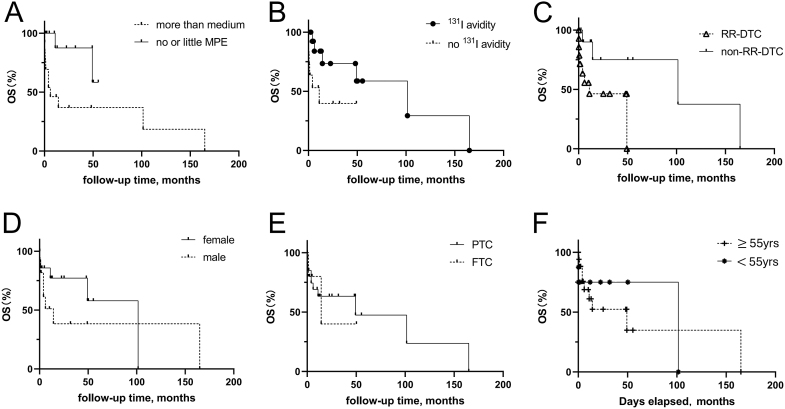
Findings of univariate analysis of factors with a potential prognostic impact on OS in patients with DTC and PM. Comparison of OS curves according to (A) level of pleural effusion, (B) ^131^I avidity, (C) RR-DTC, (D) patient sex, (E) histology and (F) age at diagnosis of PM.

Univariate analysis of the factors influencing the 5-year OS rate is shown in Table 4. Univariate analysis showed that more than a moderate amount of pleural effusion (*P* = 0.031), ^131^I avidity (*P* = 0.041) and RR-DTC (*P* = 0.030) were associated with the 5-year OS rate. The 5-year OS rate was lower in patients with more-than-moderate pleural effusion, lack of ^131^I avidity and RR-DTC than in those with no or a small amount of pleural effusion, ^131^I avidity and non-RR-DTC (Fig. 5A, B, C). Five-year OS rates were also lower in women, in those with PTC and in those aged 55 years or older at diagnosis of PM than in men, in those with follicular thyroid carcinoma and in those younger than 55 years at diagnosis of PM; however, these differences were not statistically significant (*P* > 0.05) (Fig. 5D, E, F).

**Table 4:** Clinical characteristics of 27 patients with PM from differentiated thyroid carcinoma.

Variables	Patients, *n* (%)
Sex	
Male	12 (44.44%)
Female	15 (55.56%)
Histology	
PTC	22 (81.48%)
FTC	5 (18.25%)
Age at diagnosis of PM, years	
<55	8 (29.63%)
≥55	19 (70.37%)
No. of other distant metastases	
0	0 (0%)
1	10 (37.03%)
2	5 (18.25%)
≥3	9 (33.33%)
^131^I avidity	
Yes	14 (51.85%)
No	13 (48.15%)
Concomitant distant metastasis	
No	3 (11.11%)
Lung only	20 (74.07%)
Bone only	9 (33.33%)
Both lung and bone	7 (25.93%)
Pleural effusion	
No	7 (25.93%)
Little	7 (25.93%)
Moderate	2 (7.40%)
Massive	11 (40.74%)
Time duration to discovery of PM	
Mean	6.3 months
Range	0–3 to 18.0 months
Target drug	
No	18 (66.67%)
Yes	9 (33.33%)
Progression	
No	19 (70.37%)
Yes	8 (29.63%)
Death	
No	15 (55.56%)
Yes	12 (44.44%)

PM, pleural metastasis.

## Discussion

**Figure 6:**
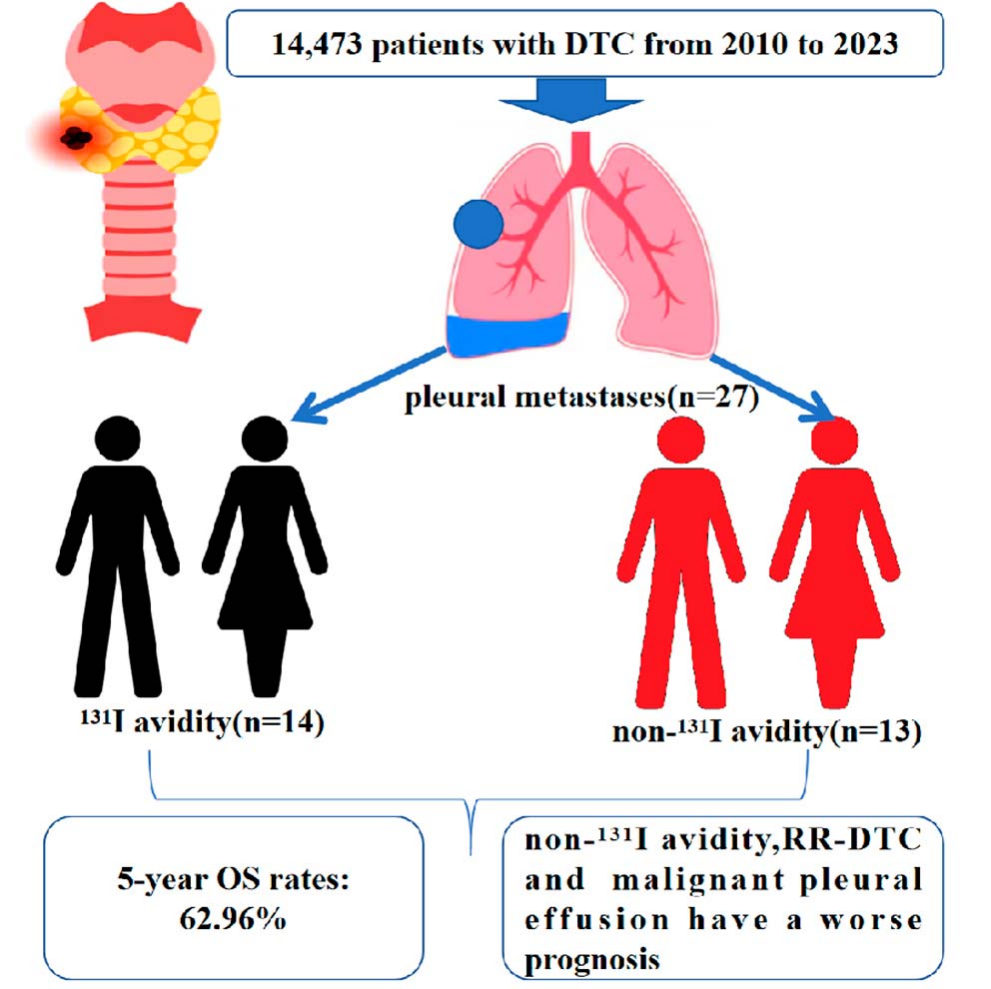
Graphical abstract illustrating the main findings.

Although the prevalence of distant metastasis from DTC has been increasing, there have been few reports on incidence, clinicopathological features, long-term outcomes and prognostic factors of PM from DTC. In this study, we investigated the clinicopathological features of 27 patients with PM from DTC after surgery and ^131^I treatment. This study is the first to focus on the long-term outcomes and prognostic factors in these patients. The incidence rate of PM from DTC in this study was 1.87‰. By the end of follow-up, eight patients (29.63%) developed PD. Twelve patients died, and 15 patients remained alive. We also identified ^131^I avidity, pleural effusion and RR-DTC to be prognostic factors in patients with PM from DTC (Fig. 6).

In this study, the 1-year, 3-year and 5-year OS rates in patients with PM from DTC were 74.07, 66.67 and 62.96%, respectively (Fig. 4A). However, in the previous literature, mortality was observed in six patients with thyroid cancer, five of whom were DTC, which are reviewed in Table 5. Three deaths were observed within the first year, four were observed within the second year, and five were observed within 5 years. The 1-year OS rate was 40.00%, which is lower than that in our present study (Fig. 4B). Possible reasons for this lack of consistency are as follows: insufficient data in the literature; the relatively small number of cases in our cohort; advances in therapy over time, such as application of ^131^I treatment and TKIs; and earlier detection as a result of regular follow-up.

**Table 5:** Prognostic factors of 5-year OS rate for patients with pleural metastasis from DTC in univariate analysis.

Variables	Patients, *n*	Log-rank	*P*-Value
Age at initial diagnosis		0.156	0.693
≥55 years	19		
<55 years	8		
Sex		1.193	0.275
Male	12		
Female	15		
Pathologic subtype		0.023	0.881
PTC	22		
FTC	5		
MPEs		4.667	0.031
None or small	14		
Moderate or large	13		
^131^I avidity		4.197	0.041
Yes	14		
No	13		
Target drugs		0.974	0.324
Yes	9		
No	18		
No. of PM lesions		0.009	0.925
≥3	17		
<	10		
RR-DTC		4.728	0.030
Yes	16		
No	11		

^131^I, radioactive iodine; FTC, follicular thyroid carcinoma; PTC, papillary thyroid carcinoma; OS, overall survival; DTC, differentiated thyroid cancer; MPEs, malignant pleural effusions.

In our cohort, the majority of the cases had concurrent metastases at other distant sites. PM is rarely the only metastatic site in patients with DTC, with only three cases (14.29%) in our cohort and three (30.00%) in the published literature (6, 7, 13). There have been 20 cases (74.07%) of the lung as the only site of concurrent distant metastasis in our study and two (20.00%) in the literature (8, 11). In the literature, PM was diagnosed in 10 patients several years after thyroidectomy with an average interval of 11.7 years (Table 5), which is approximately twice as long as that in our study (6.3 years). The longest interval between surgical resection and diagnosis of PM was 29.0 years (Table 5), which is consistent with the longest interval in our study (26.5 years). Given that PM can appear many years after thyroidectomy, long-term follow-up is important in patients with DTC (9).

The lung is the most common site of metastasis from DTC, with 5-year and 10-year OS rates of 87.0 and 69.2%, respectively (26). However, the pleura is a rare site of distant metastasis in patients with DTC. A study by Sugitani *et al.* in Japan that included 86 patients with PTC and distant metastases reported respective 5-year and 10-year survival rates of 65 and 45% (19). Combining the available data, the prognosis of PM appears to be worse than that of lung metastasis (27). PM generally occurs in patients with advanced DTC, usually with other metastases, and has a relatively poor prognosis.

The most common cytological diagnoses of malignant pleural effusion (MPE) are metastatic lung cancer, breast cancer and lymphoma (18). DTC accounts for less than 0.6% of all MPEs (6). In 2022, Mustafa *et al.* (28) showed that among 47,593 specimens, PTC was the only subtype in 15 patients with DTC involving pleural fluid. Twenty of our 27 patients had pleural effusion, with 17 being PTC. Therefore, PTC is the most common histological type of thyroid malignancy involving pleural effusion.

Pleural fluid may actively promote progression of thyroid cancer (29). MPE implies advanced systemic disease, with median survival reduced to as low as 10 months (30). The literature indicates that MPE in patients with thyroid cancer portends an extremely poor prognosis, with death typically occurring within a year after the onset of pleural effusion (28). In the literature, 10 of the patients with PM from thyroid cancer had MPE, five of whom died (Table 5). Three of these deaths occurred in the first year after the onset of pleural effusion, and the remaining two deaths occurred within the second year. In our cohort, the OS rate was significantly lower in patients with PM who had moderate or severe MPE than in those with mild or no MPE. More clinical data and specific mechanistic studies are needed.

During follow-up, ^131^I-WBS with ^131^I-SPECT/CT and ^18^F-FDG PET/CT can enhance the sensitivity, accuracy and specificity of detection of distant metastasis, aiding in early diagnosis and treatment of PD and thereby improving patient survival (31). Distinguishing primary pleural malignancy from metastasis from a radiological perspective alone is challenging (32). It is customary to use CT during follow-up, but although CT has high specificity, it has low sensitivity (29). In our cohort, 11 patients were detected by ^131^I-WBS with ^131^I-SPECT/CT and 16 were detected by ^18^F-FDG PET/CT. Furthermore, the ability of ^68^Ga-FAPI-PET/CT to detect DTC lesions was confirmed by Fu *et al.* (33). Therefore, we recommend wide application of ^131^I-SPECT/CT and ^18^F-PET/CT during the follow-up of patients with DTC to obtain the benefits of early detection.

In previous reports, the incidence of non-^131^I-avid distant metastases in patients with DTC was approximately 30% (24). In our study, distant metastatic lesions did not accumulate ^131^I in 48.15% of patients (13/27); this rate is higher than previously reported, likely because of selection bias, an inadequate sample size, the screening methods used in patients referred to our department and/or clinical management after diagnosis of DTC. ^131^I avidity is the strongest prognostic factor for survival in patients with distant metastases from DTC. Some studies have indicated that OS, progression-free survival and disease-free survival are longer in patients with ^131^I avidity than in those without ^131^I avidity (24, 34, 35), which aligns with our findings. In our study, OS was significantly longer in patients with ^131^I avidity or with non-RR-DTC.

This study had several limitations. First, it had a retrospective design and included a limited number of cases, which may have introduced bias. Nevertheless, it is the first study to evaluate the clinical outcomes of patients with PM from DTC based on diagnosis by ^131^I-SPECT/CT and/or ^18^F-FDG PET/CT. Second, we lacked uniform standards for follow-up after treatment with ^131^I. Third, disease-specific survival was not assessed because of the short duration of follow-up. Finally, other pathological features, such as *BRAF/RET* mutation and *NTRK* fusion, were not systematically evaluated during the study period.

In conclusion, this is the first report on a large group of patients with PM from DTC. The prevalence of PM was approximately 1.87%. PM generally occurs in patients with advanced DTC and may appear many years after surgery, which suggests a need for long-term follow-up. ^131^I avidity, RR-DTC and MPE were prognostic factors affecting OS in these patients. However, PM may not necessarily be a poor prognostic factor owing to regular follow-up, early detection by ^131^I-SPECT/CT and/or ^18^F-FDG-PET/CT and advances in treatment.

## Declaration of interest

The authors declare that there is no conflict of interest that could be perceived as prejudicing the impartiality of the work.

## Funding

Funding for this study was provided by the Clinical research project of Shanghai Municipal Health Commissionhttps://doi.org/10.13039/100017950 (grant number: 202340013), the Shanghai Key Discipline of Medical Imaging and National Clinical Key Specialty (Medical Imaging).

## Author contribution statement

M Liu performed the research and wrote the first draft. All authors contributed to the design and interpretation of the study and to further drafts.
